# The Evidence Base for Revascularisation of Chronic Total Occlusions

**DOI:** 10.2174/1573403X10666140331125659

**Published:** 2014-05

**Authors:** Alan Bagnall, Ioakim Spyridopoulos

**Affiliations:** 1 Institute of Cellular Medicine, Newcastle University and The Freeman Hospital, Newcastle upon Tyne, UK;; 2 Institute of Genetic Medicine, Newcastle University and The Freeman Hospital, Newcastle upon Tyne, UK

**Keywords:** Chronic total occlusion, coronary, evidence, revascularisation.

## Abstract

When patients with ischaemic heart disease are considered for revascularisation the Heart Team’s aim is to choose a therapy that will provide complete relief of angina for an acceptable procedural risk. Complete functional revascularisation of ischaemic myocardium is thus the goal and for this reason the presence of a chronic total occlusion (CTO) - which remain the most technically challenging lesions to revascularise percutaneously - is the most common reason for selecting coronary artery bypass surgery [1]. From the behaviour of Heart Teams it is clear that physicians believe that CTOs are important. Yet when faced with patients with CTOs for whom surgery appears excessive (e.g. nonproximal LAD) or too high risk, there remains a reluctance to undertake CTO PCI, despite significant recent advances in procedural success and safety and a considerable body of evidence supporting a survival benefit following successful CTO PCI. This article reviews the relationship between CTOs, symptoms of angina, ischaemia and left ventricular dysfunction and further explores the evidence relating their treatment to improved quality of life and prognosis in patients with these features.

## INTRODUCTION

Numerous studies have established that complete functional revascularisation of patients with ischaemic heart disease (IHD) results in better outcomes than incomplete revascularisation (reviewed by [2]). Chronic total occlusions (CTO) of coronary arteries are present in 15-30% of patients undergoing coronary angiography [1, 3]. They are associated with increased mortality [4-6] and are a major influence on the decision to refer for coronary artery bypass grafting (CABG) [1]. Yet complete revascularisation of viable myocardium can only be expected to relieve ischaemia if ischaemia is present and to improve the function of hibernating myocardium if its continuing dysfunction is due to chronic ischaemia. Whilst this may appear self evident, it is the basis from which all discussion of the role of CTO revascularisation must begin, since some CTOs may supply non-viable myocardium or contribute little to total ischaemic burden. This review will detail the growing evidence base that suggests that CTO PCI is an effective treatment to relieve angina, reduce ischaemia and improve survival and with its increasing success and safety profile should be regarded as an integral tool with which to achieve full functional revascularisation.

## PREVALENCE AND CURRENT STATUS OF CTO PCI

**Fig. (1):**
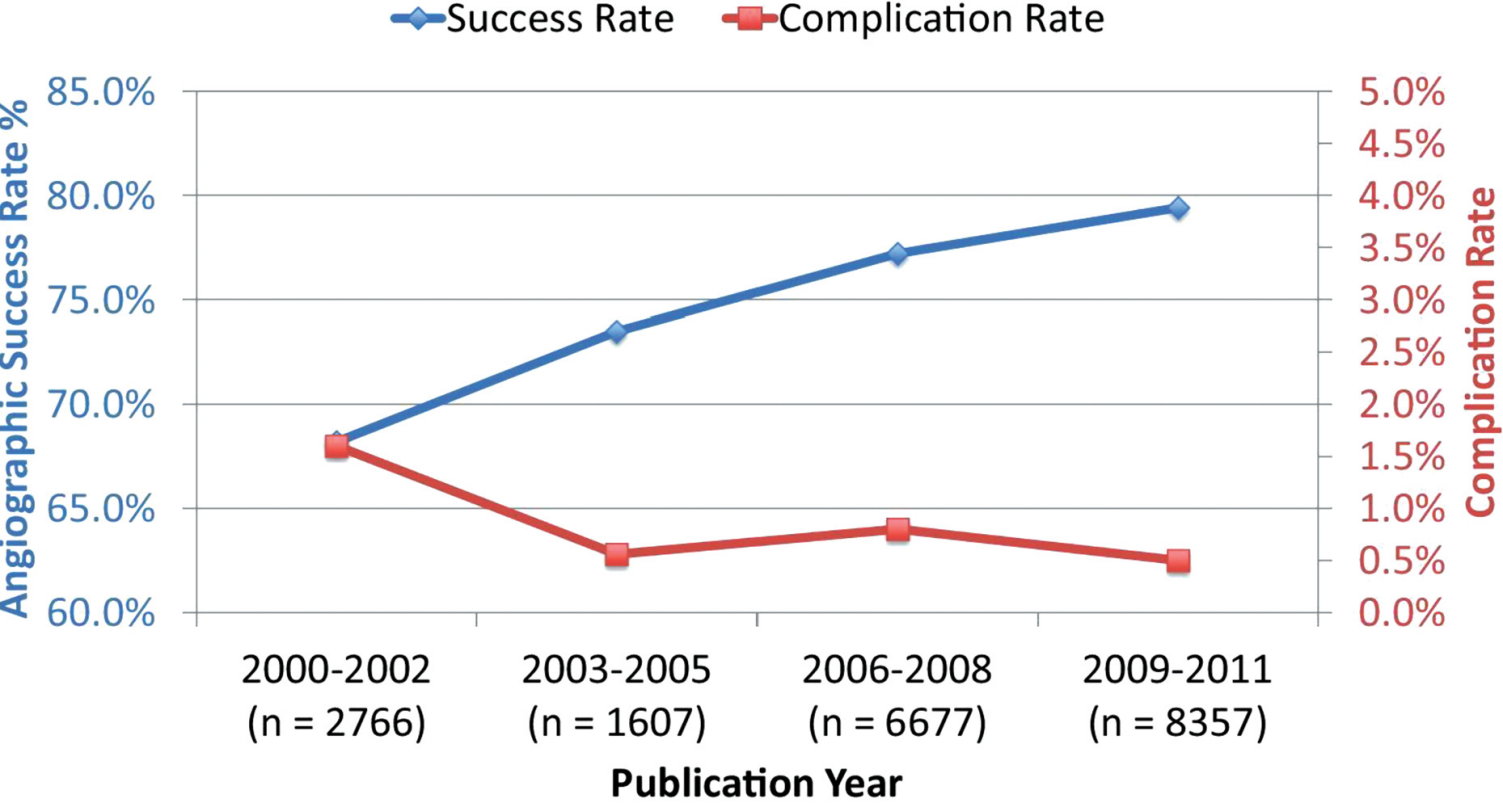
Temporal trends in cumulative angiographic success rates and major procedural complication rates, presenting according to the study publication year. Adapted from Patel VG, Brayton KM, Tamayo A*, et al.* Angiographic Success and Procedural Complications in Patients Undergoing Percutaneous Coronary Chronic Total Occlusion Interventions: A Weighted Meta-Analysis of 18,061 Patients From 65 Studies. JACC Cardiovasc Interv 2013; 6(2): 128-36.

Successful CTO PCI improves symptoms of angina [7], ischaemic burden [8], quality of life (QoL) [9], left ventricular ejection fraction (LVEF) [10-12], and survival 7, 13-16 whilst reducing need for CABG [7]. Yet despite this, only 8-15% of patients with CTO undergo PCI-based revascularisation [17-19], a figure that has remained static in the face of steady improvements in procedural safety and success (Fig. **1**). In contrast, rates of non-CTO PCI for stable IHD have increased year on year, despite a lack of evidence of improved outcomes [20]. The barriers to overcoming this paradox are numerous; lower expectation of procedural success, higher perceived risk of complications, increased technical complexity, motivation, training and catheter lab resources. But perhaps the greatest obstacle is the perception that well-collateralised CTOs are a benign condition with little relation to morbidity and that the greatest risk to the patient comes from the inherent risks of a revascularisation procedure. Unfortunately, there are no randomised controlled trial (RCT) data comparing CTO PCI to optimal medical therapy (OMT) to definitively guide practice. The available evidence will therefore be critically reviewed below. 

## CURRENT EVIDENCE

**Fig. (2):**
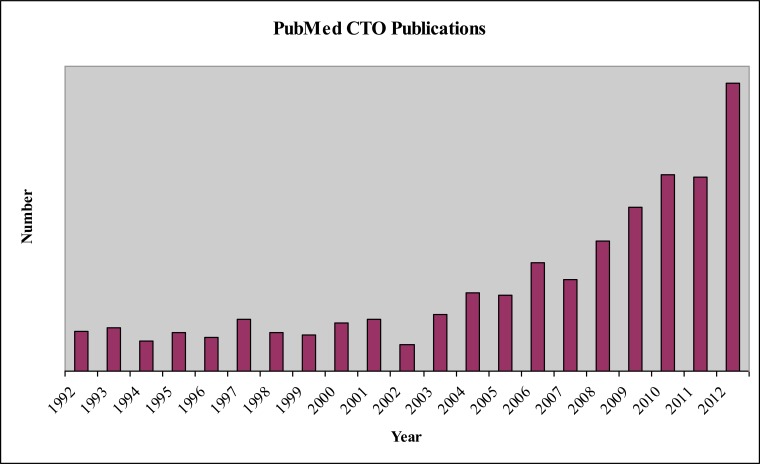
PubMed publications in the field of coronary chronic total occlusions over the last 10 years

The last 5 years have seen a significant increase in the number of publications relating to coronary CTOs (Fig. **2**). Available data has evolved from simple descriptions of short-term outcomes from single centres to large meta-analyses of registries and RCTs with robust data on procedural outcomes, LVEF, QoL and cost-effectiveness. The great majority of studies have compared successful with unsuccessful CTO PCI and it is only in recent years that the first trials specifically comparing CTO PCI with OMT have begun in earnest. At the present time, therefore, we cannot be certain that the poorer outcome observed in patients following unsuccessful CTO PCI is not simply a marker for greater patient or lesion complexity, or an adverse effect of the PCI attempt itself. On the other hand, most CTO registries do not report pre-procedural ischaemic burden or myocardial viability. As such, the benefits of successful CTO PCI may be underestimated by the inclusion of patients with no viability or little ischaema who could not be expected to benefit from a successful procedure. It is estimated that an ideal CTO PCI vs. OMT trial would require >5000 patients to adequately provide an 80% power to detect a 25% difference in the 8% 5 year mortality currently achievable with OMT [21]. The trials currently recruiting are far smaller and have thus adopted surrogate outcomes of prognosis (e.g. LVEF [22]) or non-inferiority analysis (DECISION-CTO trial; ClinicalTrials.gov identifier NCT01078051) to mitigate some of the significant challenges to completing such a trial. 

## QUALITY OF CURRENT EVIDENCE

Multiple large observational studies have reported an association between the presence of a CTO and adverse outcomes in different clinical settings, particularly following acute coronary syndromes (ACS) [6, 23-25]. This, however, does not prove that recanalisation of all CTOs will translate into more favourable outcomes. Statistical transactions such as propensity score analysis or Cox proportional hazard models may partially correct for differences between treatment and control groups for characteristics such as age, gender or LVEF. However, there will always be a strong bias towards selecting patients with the most challenging coronary anatomy, greater co-morbidity and worse prognosis *per se *for OMT, particularly in patients with CTO. The pre-procedural likelihood of successful CTO PCI is difficult to capture in a numerical parameter yet is highly likely to influence selection for attempted revascularisation. Thus, only a RCT can generate the high level of evidence necessary to establish clear guidelines. RCTs which involve comparison of invasive interventions and drugs pose additional problems to those of drug-only trials, as many patients and physicians will have strong preconceived opinions about the rights and wrongs of 'choosing' between such diverse treatment types. For example, in the context of recent non-randomised data demonstrating a survival advantage of successful CTO PCI [16], a physician faced with a symptomatic patient with a short CTO of the proximal LAD may find it difficult to recommend entering a trial that may randomise to medical therapy alone. This can lead to a trial population in which only a minority of screened patients are selected and those that are randomised have the least favourable anatomy for revascularisation. In the Freedom trial [26] comparing PCI with CABG in diabetic patients with multi-vessel disease, recruitment of 1900 patients took over 6 years from 140 centres. Pharmacology, procedural devices, stent design and even general care will have changed over this period, possibly affecting the interpretation of data. 

The American Heart Association recognises 3 levels of evidence in current guidelines (A, B and C). Level ‘A’ evidence is based on multiple RCTs or meta-analyses with multiple populations evaluated. Level B evidence (the majority of CTO data) is derived from only limited populations with non-randomised studies. Meta-analyses of these studies are attractive in terms of time and cost when no RCT data are available, yet they must be judged on the quality of the data included. Combining multiple observational studies is likely to amplify the inherent bias of each and should be regarded as hypothesis generating until RCT data are available. In the absence of a RCT (even of selected patients), the decision to recanalise a CTO should still be based on clinical judgement and the perceived health gains and risks in the individual patient. These will be reviewed below.

## CTO PCI TO IMPROVE SURVIVAL

**Fig. (3):**
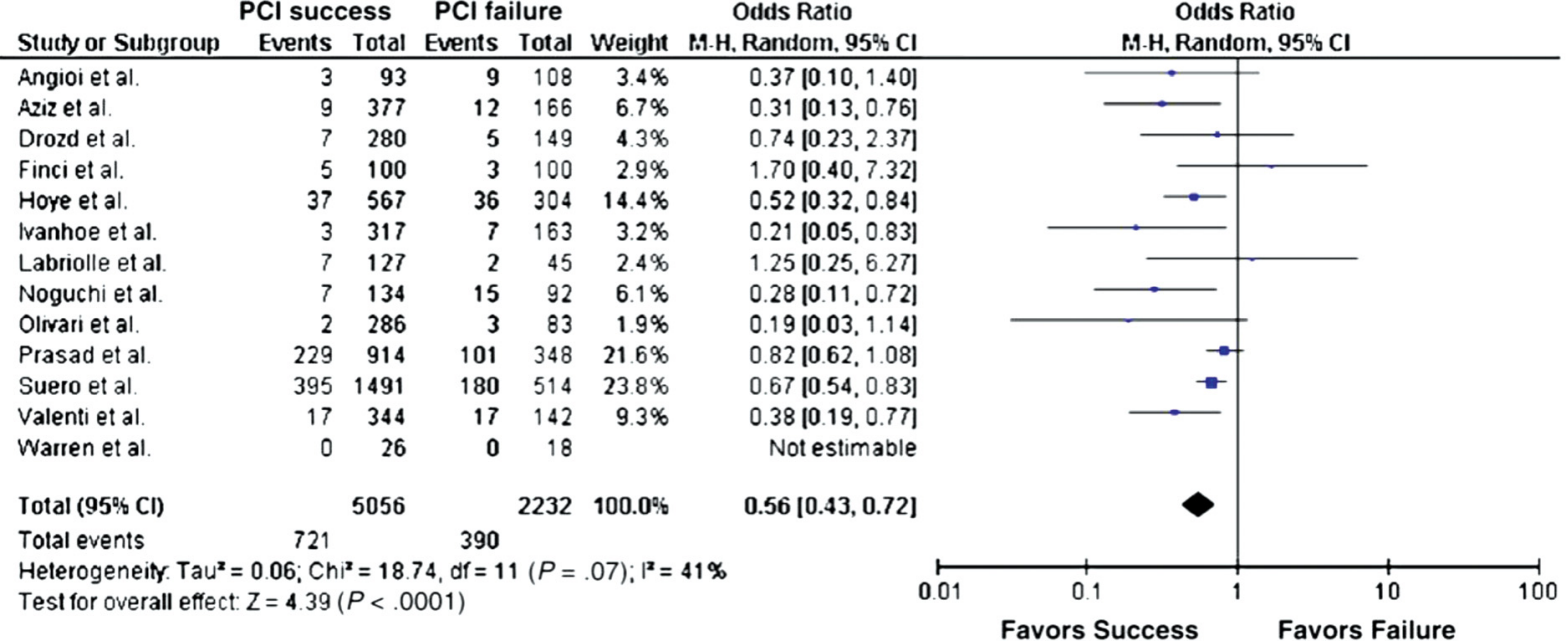
Effect of successful versus failed CTO recanalization on all-cause mortality during available follow-up. Adapted from Joyal D, Afilalo J, Rinfret S. Effectiveness of recanalization of chronic total occlusions: a systematic review and meta-analysis. Am Heart J 2010; 160(1): 179-87.

Meta-analysis of 13 studies demonstrates that successful CTO PCI reduces mortality by 44% compared to unsuccessful CTO PCI (odds ratio 0.56; 95% confidence interval 0.43-0.72; Fig. **3**) [7]. It is unclear whether LAD CTO PCI is exclusively responsible for the survival benefit with one study supporting this conclusion and another showing no association [14, 16]. However, it is important to recognise that the magnitude of treatment effect on survival of successful CTO PCI far exceeds that reported for all PCI in stable IHD and that for the observed improvement in LVEF (see below). This implies either careful selection of patients for CTO procedures (with bias against re-attempts in those with greatest co-morbidity) or harm originating from an unsuccessful attempt. If the latter were the case, then one would expect higher in-hospital or early mortality in unsuccessful cases. Five older studies report no differences in MI or urgent CABG, but a small increase in in-hospital mortality (0.02% vs. 0.006%; odds ratio 0.34; 95% confidence interval 0.18-0.65) [7]. However, a more recent study during the DES-era found no difference in in-hospital mortality or MACE between successful and unsuccessful cases [16]. A number of potential mechanisms by which revascularisation of CTOs might improve prognosis have been proposed. These include prevention of adverse left ventricular remodelling, prevention of sudden cardiac death through improved electrical stability, and greater tolerance of any subsequent ACS events. 

## CTO PCI TO IMPROVE LEFT VENTRICULAR FUNCTION

A series of small to medium-sized heterogenous observational studies have been performed that report the effect of CTO recanalisation on LVEF [10-12, 27-31]. Taken together, the results strongly suggest a modest (<5%) improvement of regional and global LV function when patients are selected on the presence of dysfunctional hibernating myocardium. The greatest recovery in LV function is thus seen in those patients with no prior history of MI, who have regional dysfunction at baseline and a patent artery at follow up. Further studies have attempted to more precisely predict functional myocardial recovery using contrast enhanced MRI, dobutamine stress ECHO, wall thickness or a combination of these different modalities. Infarction of<2 5% of the myocardial wall thickness strongly predicts recovery, >75% predicts no recovery, and between these values lies a grey zone [11, 31]. Interestingly, the presence of established functional collaterals does not predict myocardial recovery after CTO recanalisation. The formation of collaterals is dependent upon simple pressure gradients in established arterioles rather than the presence of viable myocardium [30]. 

## PREVENTION OF SUDDEN CARDIAC DEATH

The incidence of sudden cardiac death in patients with non-revascularised CTOs is 5 times greater (2.7% vs. 0.5%) than in those with successful revascularisation [32]. Animal models (although imperfect) have provided insight into potential mechanisms for this. Sudden cardiac death is common in a porcine model of chronic total LAD occlusion (49% mortality at 5 months) and is caused exclusively by ventricular fibrillation (VF) precipitated in over 80% of cases by a preceding episode of ventricular tachycardia (VT) [33]. Like humans, viable hibernating myocardium in the territory supplied by the occluded LAD in the pig is reliant upon a collateral blood supply. However, no histological evidence of acute vascular occlusion or significant new infarction was seen to implicate a sudden ischaemic event in the initiation of VT or VF. Whilst it is well recognized that a discrete scar predisposes to arrhythmia, these observations suggest that cellular adaptations in response to chronic repetitive ischaemia in viable hibernating myocardium, along with inhomogeneity of sympathetic activity, may also render the heart vulnerable to fatal arrhythmias [33, 34]. Clinical trials in humans are consistent with this hypothesis; the presence of a CTO in patients with LV dysfunction is associated with greater arrhythmia burden, risk of death and is an independent predictor of appropriate ICD therapy (H.R. 3.5). Further, ICD therapy improves survival in medically treated patients with LV dysfunction at high risk of ventricular arrhythmias [35] but not after surgical revascularisation [36].

## INFLUENCE OF NON-INFARCT-RELATED CTOs IN PATIENTS WITH ACS

CTOs of a non-infarct-related artery (IRA) are present in 7-13% of all patients with STEMI [37-39] and in 21% of those with diabetes [4]. Multiple observational studies of STEMI 4-6, 23, 24, 37, 38, 40-42 and NSTEMI [25] patients have now identified the presence of a non-IRA CTO as an independent predictor of significantly higher early and late mortality. Indeed the majority of the adverse impact of multi-vessel disease in STEMI is due to the presence of a concurrent CTO [4, 23, 24]. 

Cardiogenic shock remains the most important predictor of early mortality in STEMI. Over 25% of patients who present with cardiogenic shock have at least 1 CTO of a non-IRA. 30 day mortality rates in shock patients of 100%, 65% and 40% for those with >1CTO, 1 CTO or no CTO respectively, have been reported [40, 42]. After left main stem occlusion (H.R. 6.55), the presence of a non-IRA CTO is the most important independent predictor of cardiogenic shock (H.R. 4.2) and after adjustment for LVEF and renal function remains an independent predictor of 30 day mortality [42]. 

CTOs are associated with impaired microvascular perfusion [43] and it is perhaps unsurprising, therefore, that a non-IRA CTO also predicts impaired myocardial reperfusion [23, 39, 44] following primary PCI with an associated increase in infarct size and mortality [39]. As a result of less successful treatment, surviving patients are nearly twice as likely to have a LVEF of ≤40% immediately after STEMI and follow up at 1 year demonstrates further declines in LVEF [38] due to adverse remodelling. Taken together, these data suggest that patients with CTO tolerate ACS less well at presentation, derive less benefit following reopening of the IRA, and those that survive fair worse in the longer term.

Anecdotal evidence exists that prior opening of a CTO can help preserve LV function through reverse collateral flow into a previous donor vessel during acute MI [45]. This may be one additional mechanism through which CTO PCI improves tolerability of contralateral ACS events. However, the on-going prospective randomised controlled EXPLORE trial [22] will be the first to examine definitively whether recanalization of a non-IRA CTO early after STEMI can improve LVEF, perhaps the most important prognostic determinant of late survival and clinical events. Importantly, this trial will randomise patients 1:1 to early CTO-PCI or OMT.

## CTO PCI TO IMPROVE SYMPTOMS

The majority of CTO PCI procedures are performed to relieve symptoms of angina. Early studies comparing balloon angioplasty and bare metal stents (BMS) confirm its efficacy in this regard, with angina free status achieved in 57-90% of cases at medium and long term follow up [46-48]. Two year follow up after drug eluting stents (DES) reveals that only 15.1% of patients had residual angina/silent ischaemia at 2 years compared with 32.1% of patients following BMS [49]. Six studies have reported angina status after successful compared to unsuccessful PCI, 4 of which were from the balloon-only era. A 55% relative risk reduction in residual/recurrent angina favouring CTO-PCI was seen [7]. In the stent studies (all BMS) [50, 51] successful cases were more likely to be angina free at 1 year (88 % vs. 75-80%). The interpretation of these data, however, is clouded by a higher incidence of subsequent CABG in the unsuccessful group (8.1-15.7% vs. 2.4-3.6%) which may have reduced the differences in symptoms between groups, and by a lack of reported use of medical therapy. 

Considering symptom relief is the major indication for revascularisation, there are few studies of the impact of CTO PCI upon QoL and none comparing treatment with OMT. Using the Seattle Angina Questionnaire (SAQ), Grantham and colleagues measured health status at baseline and 1 month post-PCI in patients with either ≥ class II angina or significant ischaemia [9]. In common with the majority of CTO studies, comparison was made between those with successful and unsuccessful procedures. Successful CTO PCI reduced angina frequency (9.5 point change), improved physical function (13.1 point change) and improved QoL (20.3 point change). In contrast, unsuccessful PCI did not change subjective health status. The magnitude of change in health status was similar to that achieved after non-CTO PCI [52, 53] or CABG [54]. As might be expected, previously asymptomatic patients saw no significant improvement in angina frequency or physical limitation scores after successful intervention. However, overall QoL did improve modestly in previously asymptomatic patients, though the placebo effect of knowing a medical intervention has been successful is difficult to exclude. In a study of patients after successful LAD CTO PCI [55] an improvement in angina frequency, SAQ QoL and 6 minute walking distance was also observed at 1 year in those with either a reversible or fixed anterior wall defect on pre-procedural SPECT. However, no health status benefit was observed in those patients with no ischaemia at baseline. Taken together, these QoL data clearly illustrate the substantial negative effect upon health status of CTOs. When patients are selected for revascularisation on the basis of symptoms or objective evidence of significant ischaemia, a beneficial effect can be expected from a successful procedure.

## CTO PCI TO RELIEVE ISCHAEMIA

A proportion of patients with CTOs have minimal or no symptoms of angina and in such instances revascularisation decisions must be based on an expectation of a prognostic benefit. The presence and extent of inducible ischaemia has been proposed as an important predictor of adverse cardiac events for patients with stable IHD [56-58]. Although it may seem self-evident that a blocked artery supplying a viable area of myocardium will result in ischaemia, it is often argued that a well developed collateral supply to the distal vessel is sufficient to meet the metabolic demands of the myocardium and prevent clinical events. The evidence that supports the hypothesis that CTOs cause ischaemia and that ischaemia precipitates clinical events will be reviewed. 

## DO PATIENTS WITH WELL COLLATERALISED CTOs EXPERIENCE ISCHAEMIA?

It is commonly assumed that well collateralised CTOs do not produce severe ischaemia to the same extent as a sub-totally stenosed coronary artery. Measurement of fractional flow reserve (FFR) is now regarded as the gold standard invasive modality for identification of inducible ischaemia and for guiding revascularisation decisions [59]. FFR is related to both the severity of coronary stenosis and the mass of tissue perfused. Let us first consider the collateral donor artery supplying viable myocardium in a CTO territory. It must clearly perfuse a greater mass of tissue than normal and this has important physiological implications if there is any degree of stenosis in the donor artery. Re-opening a CTO will clearly improve flow in the occluded vessel, but by also reducing the myocardial mass supplied and hence the hyperaemic flow in the donor artery, it can also prevent ischaemia in the donor territory [60, 61]. Indeed, collateralisation of a CTO is an independent predictor of FFR in donor vessels and is associated with significantly lower FFR values [62]. 

If we now consider the myocardium distal to the CTO, can a well-developed contralateral collateral circulation provide an adequate blood supply during hyperaemia? The first study to invasively examine this question made direct measurement of perfusion pressure in the collateralised CTO distal vessel segment during CABG surgery [63]. This demonstrated that the haemodynamic influence of a well collateralised CTO was equivalent to that of a 90% upstream epicardial stenosis, well in excess of currently accepted angiographic criteria for revascularisation. These studies have subsequently been replicated during PCI by measurement of FFR and Doppler flow velocity. Werner and colleagues established that the majority of donor collaterals are sufficient to prevent ischaemia under rest conditions. However, collateral fractional flow reserve (FFR_Col_; analogous to FFR for epicardial arteries) during adenosine hyperaemia was only 0.32±0.13 (range 0.03-0.78), confirming that collaterals are unable to prevent ischaemia during stress [64]. In one third of patients there was additional evidence of coronary steal by the donor artery that may further reduce collateral functional reserve.

## WHAT IS THE ASSOCIATION BETWEEN ISCHAEMIA AND CLINICAL EVENTS IN STABLE IHD?

The ability of stress radionucleotide imaging to predict adverse events based on the extent of ischaemia is well established [65]. In symptomatic patients with functionally non-significant coronary stenoses treatment with OMT is associated with a low incidence of MI or death (<1%/year). In contrast, functionally significant stenoses have an event rate of 5-10%/year if treated medically (reviewed by [59]). The nuclear sub-study of the COURAGE trial [66] found that a significant reduction in ischaemia by either OMT or PCI was associated with reduced clinical events, whilst registry data demonstrates that a ≥5% worsening of ischaemia during 5 year follow up is an independent predictor of death or MI [67]. In asymptomatic patients a threshold of ischaemia involving ≥7.5% of the myocardium was associated with a cardiac death or MI rate of ≥3%/year [68]. Controversy remains over whether there is a specific advantage to ischaemia reduction by revascularisation with some studies showing no difference in treatment modality [69] and others favouring revascularisation [67, 70]. Although meta-analysis of 13 studies of successful vs. unsuccessful CTO-PCI demonstrated significant reductions in mortality, referral for CABG and angina, there was no benefit of CTO PCI for the reduction of MI [7]. The significance of this observation is uncertain in the absence of details of the site of infarction (since re-opening a CTO would not be expected to prevent MI caused by *de novo* plaque rupture in a different artery) or the incidence of stent thrombosis (ST) in the CTO vessel and other non-CTO arteries revascularised at the same time. 

## DOES CTO PCI RELIEVE ISCHAEMIA MORE EFFECTIVELY THAN OPTIMAL MEDICAL THERAPY?

There are no RCTs or observational studies comparing planned OMT with CTO PCI. Rather, all available studies have compared outcomes with patients who had a failed initial strategy of CTO PCI. None have reported details of post-procedural medical therapy use by the different groups which further limits their interpretability [7]. However, assessment pre- and 1 year post-CTO PCI reveals a reduction in ischaemic burden [55] by an average 6 percentage points [8, 71] whilst unsuccessful cases saw no change in ischaemic burden [71]. In non-CTO studies comparing OMT with OMT+PCI in stable CAD, PCI was associated with a greater reduction in the extent of myocardial ischaemia (mean reduction -2.7% vs. -0.5%) and a greater proportion of patients who achieved a significant (≥5%) reduction in ischaemia (33% vs. 19%) [66]. This was particularly evident for patients with moderate to severe pre-treatment ischaemia (78% vs. 52%) though no difference in outcome according to treatment allocation was seen [69]. Registry data suggests that OMT may be less able to protect against ischaemia in long term follow up. A study of 1425 patients with documented CAD from the Duke nuclear cardiology registry were treated with OMT, PCI or CABG and underwent serial nuclear imaging over a 5 year period [67]. Fifteen percent of patients treated with OMT developed ≥5% worsening of ischaemia compared with 6.2% of PCI patients and 6.7% of CABG patients. Worsening ischaemia was an independent predictor of death or MI (H.R. 1.6). The results of the randomised ISCHEMIA trial (ClinicalTrials.gov identifier: NCT01471522) are now awaited and should provide the definitive answer to the question of optimal initial management strategy for patients with moderate to severe ischaemia irrespective of coronary anatomy. For the time being, available evidence suggests that CTOs supplying viable myocardium cause ischaemia and that any incremental benefit of CTO PCI over OMT will likely be greatest in those with the greatest extent of inducible ischaemia at baseline.

## COST EFFECTIVENESS OF CTO PCI

The economics of modern health care demand that any treatment be both effective and cost effective. In the absence of any RCT data to support a prognostic impact, the cost effectiveness of CTO PCI to improve angina and QoL has been estimated [72]. A decision-analytic model was tested using assumptions based on the estimated costs of CTO procedures, rates of re-intervention, typical rates of successful CTO PCI and predicted complications taken from 16 observational studies, along with QoL outcomes from The Flowcardia’s Approach to Total Occlusion Recanalisation (FACTOR) Trial [9]. Over 5 years in a typical reference patient aged 60 with class III-IV angina, the cost of CTO PCI was US$3707 more expensive than OMT, but the QALY gain (2.38 vs. 1.99) was also greater, equating to a cost of US$9505/QALY, well below the US$50 000/QALY benchmark. CTO PCI became cost effective in the model after only 2 years and in simulations assuming differing costs and efficacy rates remained cost-effective in 60% of scenarios at a willingness-to-pay (WTP) threshold of US$50 000/QALY and 50% of scenarios at a WTP threshold of US$18 000/QALY. Although not directly related to procedural cost-effectiveness, it is important to note that the longer duration of many CTO procedures has the effect of reducing patient turnover through the catheter laboratory. For health economies which pay a fixed sum per revascularisation procedure regardless of time taken or additional equipment used, CTO PCI may have a significant adverse impact on income generation for hospitals. 

## SAFETY AND OUTCOME OF CTO PCI

### Durability of CTO PCI

Any assumption that CTO PCI has a durable influence on clinical outcomes must assume that arterial patency is maintained in the long term. Identification of factors predictive of early re-occlusion may, therefore, be helpful. There are few studies with mandated angiographic follow up of all cases. The largest, a registry of over 800 successful CTO cases with 6-9 month angiographic follow in 82%, reported a re-occlusion rate of 7.5% [73]. Meta-analysis of 4394 patients included in smaller registry studies and RCTs with ~75% angiographic follow up suggest that DES use can significantly reduce both re-occlusion (3-4% vs. 10-14% after BMS) and non-occlusive angiographic re-stenosis (11% vs. 37%) [74]. Overall, the risk of restenosis following DES for CTO (11%) [74] is of similar magnitude to that seen when DES are used in other off-label indications [75]. In the only RCT to report individual MACE outcomes (PRISON-II) [76], DES use was associated with a 55% relative risk reduction (RRR) in MACE compared to BMS, predominantly due to a 59% RRR in target vessel revascularisation with no difference in mortality, stent thrombosis or MI seen [74]. Use of the STAR technique was strongly predictive of re-occlusion (57%) but there were no cases of re-occlusion when limited sub-intimal and re-entry techniques for retrograde approaches were used [73]. 

### Safety of CTO PCI

The safety and success of CTO PCI has increased steadily over the last decade [77]. However, one of the key barriers to referral remains a perception of increased peri-procedural complications compared to non-CTO PCI. In fact, a recent meta-analysis of 65 CTO PCI studies including more than 18,000 patients over an 11 year period demonstrated an overall MACE rate of 3.1%, comprising a 0.2% risk of death, 0.1% risk of emergency CABG, <0.01% risk of stroke and a 0.2% risk of Q-wave MI (2.5% risk for any cardiac biomarker rise) [77]. Definite ST was seen in 0.4-1.28% of cases [73, 74] and definite/possible ST in 1.99% [74]. Coronary perforation was relatively common (2.9%), but led to tamponade in only 0.3% of cases [77]. Contrast-induced nephropathy complicated 3.8% of cases. Recorded radiation injury was extremely rare (<0.01%) though diagnosis and data capture up of such complications are often problematic. A direct comparison of all UK PCI data from 2011 indicate that the risk of single vessel CTO PCI in the modern era is now only marginally greater than that of stable single vessel non-CTO PCI (risk of in-hospital death 0.17% vs. 0.15%, emergency CABG 0.07% vs. 0.04%, stroke 0.07% vs. 0.03%, Q-wave MI 0.03% vs. 0.08%) and less than that of PCI for saphenous vein grafts (SVG; in hospital death 1.4%, Q-wave MI 0.4%) [78]. Indeed, SVG PCI still accounts for 5-10% of all PCIs [79] despite no evidence of improved survival [79, 80], poorer long term patency, and worse 30 day mortality compared to CTO PCI (2.7% vs. 0.42% [78]). 

### What are the risks of a failed CTO PCI?

Meta-analysis of current data demonstrates a ~77% success rate for CTO PCI [77], though high volume operators using hybrid approaches can now achieve success rates of ~90% [81, 82]. Complication rates of all types during CTO PCI are significantly higher in failed cases compared with successful cases with an attendant increase in overall mortality (0.42% vs. 1.54%) [77]. This difference may indicate that at least some of the survival advantage reported in studies comparing successful with unsuccessful CTO PCI is due to the harm caused by a failed PCI attempt. However, the mortality of patients with unsuccessful CTO PCI who are subsequently managed medically is significantly higher than those with unsuccessful CTO PCI who go on to have CABG [16]. This suggests that comorbidities present in unsuccessful cases that disincline physicians to refer for further PCI attempts or for surgery may also contribute to the excess mortality. 

**Fig. (4):**
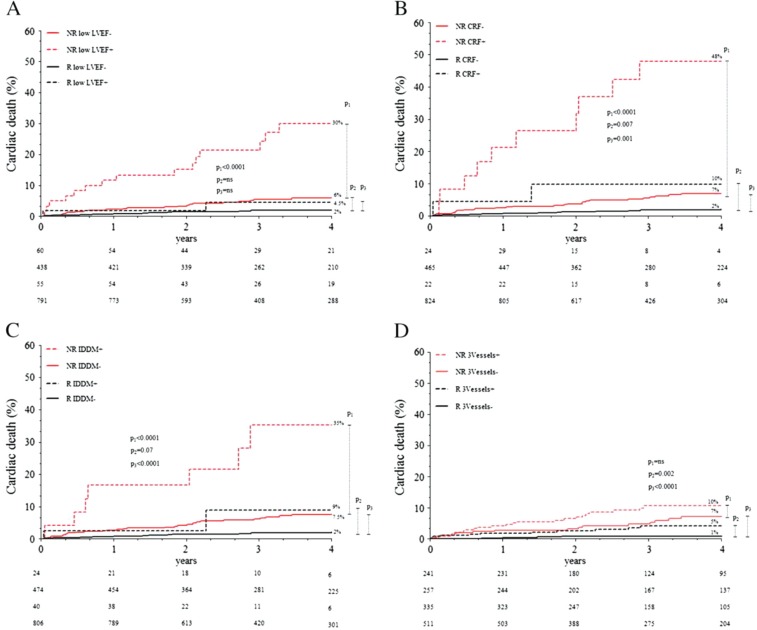
Kaplan–Meier analysis of cardiac death incidence in Revascularized (R) and Not revascularized (NR) CTO patients stratified by the presence (+) or absence (-) of low Left Ventricular Ejection Fraction (LVEF, **panel A**), chronic renal failure (CRF, **panel B**), Insulin-Dependent Diabetes Mellitus (IDDM, **panel C**) and coronary 3-vessel disease (3Vessels, **panel D**). Adapted from Godino C, Bassanelli G, Economou FI*, et al.* Predictors of cardiac death in patients with coronary chronic total occlusion not revascularized by PCI. Int J Cardiol 2013.

Despite the low overall complication rate, a large proportion of patients with a symptomatic CTO will never have revascularisation attempted. Those least likely to ever undergo a PCI attempt are older patients, those with multiple CTO lesions and those with no prior CABG [32]. If we take into account the procedural risks and potential benefits of CTO PCI, what are the risks to patients of *never* attempting revascularisation? In a longitudinal study of 1345 CTO cases by Godino and colleagues [32] no difference was found in mortality between failed attempted CTO-PCI patients and those never attempted. Over 4 years of follow up, poor LV function, chronic renal failure and diabetes each independently predicted at least a four-fold higher risk of death in non-revascularised CTO patients. In contrast, there was no difference in the prognosis of CTO patients with, compared to without these comorbidities if revascularisation had been successful [32] (Fig. **4**). The greatest benefit of CTO revascularisation would thus appear to be in patients with these high risk clinical features and careful consideration should be given to repeated or alternative revascularisation attempts in such cases.

## COMPARISON OF CTO PCI WITH SURGICAL REVASCULARISATION

Approximately 25% of patients with CTO identified at coronary angiography undergo CABG, with the CTO vessel successfully grafted in around 90% of cases [83, 84]. In a contemporary US surgical registry, 42% of all CABG operations involved a CTO vessel with RCA CTOs the most common (48%) [84]. These data suggest that the presence of a CTO is an important factor in guiding the mode of revascularisation decision by the Heart Team. Indeed, in the SYNTAX trial the proportion of patients with a CTO in the CABG registry was more than double that in the overall trial population [85].

However, like CTO PCI, bypass grafting of a CTO vessel presents several unique technical challenges that may adversely influence short and long term patency. Identification of the anastomotic site pre-operatively may be problematic because of poor visualisation provided by the collateral circulation [86]. This may lead to more distal graft placement, as can the diffuse pattern of coronary disease observed in CTO patients. Diffuse disease also increases the need for endarterectomy or plaque excision [87]. Patency of LAD CTO grafts is 100% after 1 year [88] but in the PRAGUE-4 study was only 23% in other territories [88], despite excellent early results [89]. In contrast, re-occlusion of CTOs after successful PCI occurs in only 3% of cases treated with DES [73, 74]. The available data thus supports use of PCI as the preferred treatment for RCA and Circumflex CTOs in the absence of multi-vessel disease, particularly for those who have previously undergone CABG [90]. Indeed, around 15% of all CTO PCI is now performed on patients who have previously had CABG [77].

## CTO TREATMENT WITHIN THE SYNTAX TRIAL

In the SYNTAX trial of CABG vs. PCI in patients with multi-vessel disease, 27% of patients within each treatment arm had at least 1 CTO. CTO patients had more diabetes, more commonly had 3 vessel disease, left main disease, diffuse disease and calcified vessels and as a result had higher mean SYNTAX scores (32 vs. 23 in the non-CTO cohort). CTO treatment success rate was 68.1% in CABG patients and 49.4% in PCI patients (well below the success rates reported in current registries [77]). Complete revascularisation was only achieved in 49.6% of the CABG arm and 35.8% of the PCI arm, suggesting additionally that many CTO (or other) lesions were not bypassed or percutaneously attempted. This likely reflects the contribution of small, perhaps clinically insignificant vessels to the overall SYNTAX score. At 12 months, there was no difference between CABG and PCI CTO groups in the rate of death, MI, stroke or symptomatic ST or graft occlusion, but overall MACE was higher in the PCI group due to more frequent need for repeat revascularisation. Highlighting the safety of CTO PCI, there was no difference in MACE rate between CTO PCI patients and non-CTO PCI patients.

## CONCLUSIONS

Recent advances in training, technique and novel dedicated devices have increased the success rates of CTO PCI. At present, data from single centre studies and meta-analyses suggest that successful CTO PCI performed at experienced centres improves symptoms, ischaemia and survival in selected patients compared to those with failed attempts. Current evidence falls short of the quality required to provide clear recommendations for all patients, particularly as no direct comparison of CTO revascularisation with a primary strategy of OMT in patients suitable for either treatment has been made. There remain considerable barriers to performance of adequate RCTs, including availability of the necessary expertise to achieve optimal CTO PCI success rates and the recruitment of patients who are ideal candidates for CTO PCI yet are willing to be treated medically. Recruitment criteria (angiographic only vs. prior quantification of ischaemic viable myocardium) and end-points for such trials will also prove challenging. However, a RCT will likely be required if there is to be a sea change in the management of CTO patients.
